# Origin of relationship between ferromagnetic response and damage in stretched systems

**DOI:** 10.1038/s41598-018-32149-z

**Published:** 2018-09-12

**Authors:** S. Merabtine, F. Zighem, A. Garcia-Sanchez, V. Gunasekaran, M. Belmeguenai, X. Zhou, P. Lupo, A. O. Adeyeye, D. Faurie

**Affiliations:** 1 LSPM-CNRS, Université Paris XIII-Sorbonne Paris Cité, 93430 Villetaneuse, France; 2 Information Storage Materials Laboratory, Department of Electrical and Computer Engineering, National University of Singapore, Singapore, 117576 Singapore

## Abstract

This article presents a study whose purpose is to elucidate the damage effects in thin films on their magnetic response. Co_40_Fe_40_B_20_ and Ni_80_Fe_20_ films of different nanometric thicknesses were stretched by more than 10% and *in situ* probed by atomic force microscopy measurements to determine their irreversible mechanical behavior (multi-cracking, buckling). Once these phenomena have been well identified, magnetic behavior of these stretched systems has been studied by ferromagnetic resonance to measure resulting magnetic anisotropy and damping evolutions. All of these experimental studies show that the magnetic properties are mainly affected by the stresses generated during the damage but not by the local discontinuities induced by the numerous cracks and buckles. This is in particular confirmed by the almost zero sensitivity to the damage of the magnetic properties of Ni_80_Fe_20_ alloy which is known for its vanishing magnetostriction.

## Introduction

Nanoscale systems fabricated on flexible or stretchable substrates (elastomers or polymers such as Kapton®, PET, PDMS, PVDF, etc.) are being studied more and more because of their ability to adapt to non-planar surfaces, particularly in confined environments^1–3^. In addition, these systems have the advantage of being lighter and less expensive than their counterparts deposited on more conventional rigid substrates (silicon, …). In recent years, many magneto-electronic devices have been made on different polymer substrates^4–7^. The ability of these magnetic thin films on polymer substrates to be folded or stretched is essential^8–12^, but their use is still delicate, which can be a brake on the industrialization of these systems^13^.

The main issues are to understand how the applied strains to the flexible magnetic systems impact their magnetic properties^14–16^. Obviously, when a thin film is deposited on a flexible substrate, it is usually submitted to high mechanical stresses due to the stretching or the curvature of the whole system. Moreover, these stresses are all the more important given that the Young’s modulus contrast is high between the film and the substrate. This contrast is characterized by the Dundurs parameter *α*_*D*_^17,18^:

$${\alpha }_{D}=\frac{\overline{{Y}_{f}}-\overline{{Y}_{s}}}{\overline{{Y}_{f}}+\overline{{Y}_{s}}}$$

were $\overline{Y}=Y/(1-{\nu }^{2})$ is the plane strain elastic modulus^19^, *ν* is the Poisson ratio, subscript *f* and *s* refer to the film and the substrate respectively. The value of *α*_*D*_ varies from −1 for a rigid substrate to +1 for a compliant substrate. In the last case, a small effort applied to the system lead to high stresses in the thin film.

These stresses may have an important effect on the static and dynamic magnetic properties of thin films on polymer substrates. In particular, it is important that the large deformations to which they are subject are not harmful to their functional properties. In fact, beyond the classical magnetoelastic effects observable at small strains, the phenomenon of cracking^20,21^ and associated localized buckling observed for inorganic thin films on organic substrates tensily stressed^22–25^ lead to heterogenous strains that may have effects on magnetic properties. However, these are rarely discussed and have never been studied in depth.

In this work, we focused on experimental identification of cracking mechanisms for two magnetic alloys (Co_40_Fe_40_B_20_, Ni_80_Fe_20_) deposited on Kapton® substrate (125 mm). The phenomena of multi-cracking but also buckling of thin films have been studied. Thin films surface was probed by atomic force microscopy (AFM) during or before tensile tests to clearly identify these mechanisms. Beyond the mechanical properties, we then examined the magnetic properties of these systems. At first, we estimated magnetoelastic properties of the two materials by piezoelectric actuation and *in situ* ferromagnetic resonance (FMR). These data collected at small strains are important for interpreting results for higherstrains. Subsequently, we have identified the *ex situ* effects of irreversible phenomena, particularly “cracks” and “cracks-buckling” regimes, on magnetic properties of thin films (anisotropy and damping).

## Experimental Section

### Materials deposition

Co_40_Fe_40_B_20_ (CoFeB thereafter) and Ni_80_Fe_20_ thin films were deposited on 125 m thick Kapton® substrates. In order to avoid any contamination, cleaning of these substrates was carried out in a clean room. They were immersed in isopropanol in a beaker and placed in an ultrasonic bath for 10 minutes. Following this, they were cleaned using distilled water and a nitrogen gun, before being stored in a clean plastic case. Samples were made by varying the thicknesses of the thin films from 10 to 100 nm. On the other hand, the substrates were once again cleaned with a plasma etching (Ar + O_2_) at 3 mTorr for 120 seconds just before the deposition. The deposits were made under vacuum (residual pressure of 2.10^−8^ Torr) at an argon pressure of 1 mTorr. This is to eliminate any organic residues and improve the adhesion of the films to the substrate. CoFeB material is known to be amorphous at room temperature and is very used in spintronics applications^26^, while Ni_80_Fe_20_ is a reference crystalline material for magnetism community because its magnetoelastic constants are close to zero^27^.

### *In situ* mechanical testing

The tensile tests have been performed by using a technique combining a micro-tensile tester and AFM observations^28,29^. The tensile loads were applied to specimens by means of a 300 N Deben^TM^ tensile module. This tensile tester is equipped with a 300 N load cell enabling the force measurement with a precision of 0.1 N. The strain rate has been kept constant, equal to 3.10^−4^ s^−1^ during loading ramps (up to 20%, the initial length is 28 mm and the displacement rate is 0.5 mm/min). Samples are mounted horizontally, clamped to a pair of jaws and supported on stainless steel sliding bearings. A dual threaded lead screw drives the jaws symmetrically in opposite directions, keeping the sample centered in the field of view. The module is ideally suited for use with AFM. The AFM observations have been made during test breaks by using a standard Veeco D3100 microscope (in contact mode).

### Ferromagnetic resonance (FMR)

The FMR setup allows the determination of the resonance field *H*_*res*_ of the uniform precession mode by sweeping the applied magnetic field in presence of a fixed pumping radio frequency field ${\overrightarrow{h}}_{rf}$ (i.e. microwave driving frequency *f*)^30^. In order to enhance the signal to noise ratio, a weak modulation of the static applied magnetic field (here 5 Oe at 175 Hz) is performed. Thus, in absence of applied strains, the resonance field of the uniform precession mode is expected to only depends on the gyromagnetic factor *γ*, the saturation magnetization *M*_*s*_ and the presence of magnetic anisotropies. The presence of in-plane anisotropies can be easily detected by measuring the in-plane angular dependence of the uniform precession mode. Finally, the magnetoelastic properties of the films have been studied using the FMR setup with *in situ* micro-mechanical testing (at small strains, *e.g*. about 0.1%)^31^. For this purpose, the film/substrate systems have been glued on top of piezoelectric actuators. The principe of this *in situ* measurements is to deform the films through the voltage applied to the actuator, the strains being perfectly transmitted from the actuator to the films. The resonance fields are measured by FMR while the strains are measured by digital image correlation (DIC) from optical tracking of the mottled back surface^32,33^.

## Experimental Results and Discussions

### Mechanical properties

**Figure 1:**
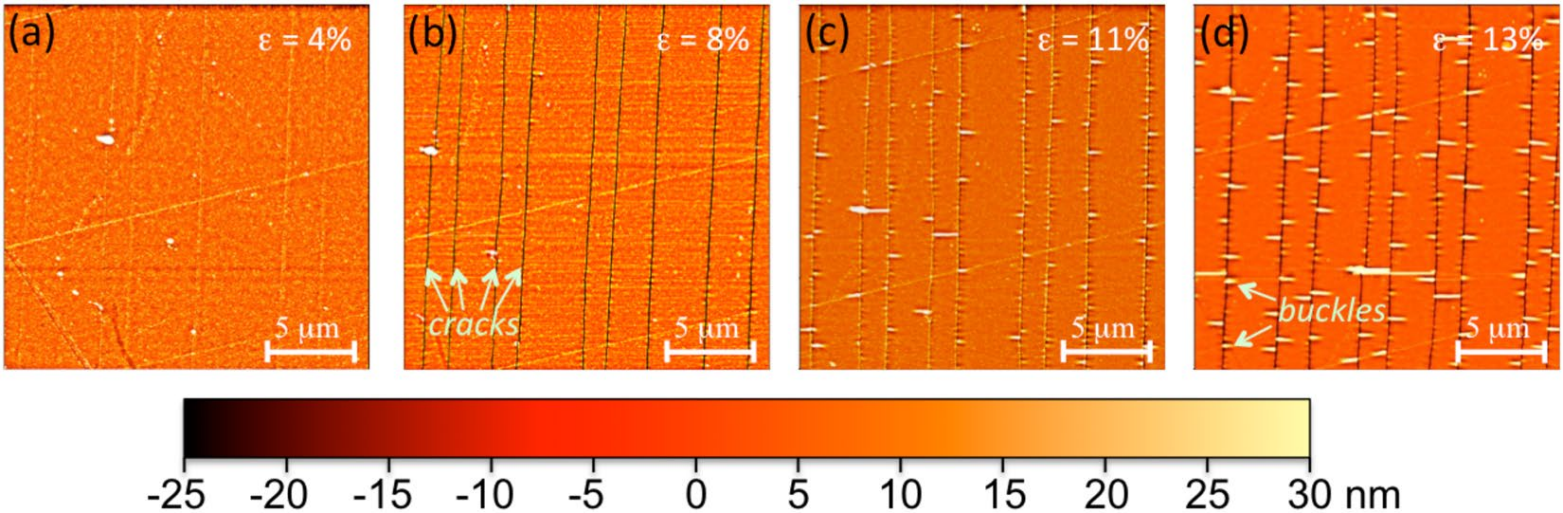
AFM images highlighting the multi-cracking and subsequent buckling of a CoFeB 20 nm-film obtained at different strain states.

In this section, we focuse on experimentally identifying cracking mechanisms for the two studied alloys (CoFeB and Ni_80_Fe_20_) deposited as thin films on Kapton® substrate (125 mm). Figure 1 shows images of the surface of a 20 nm thick CoFeB thin film for four applied strain states in order to understand the cracking mechanism. The goal here is to identify the cracks morphology that appear within the films. We tried to record our images on the same region of the film although drifts of a few micrometers are inevitable in that feedback loop is not available on the microscope. It is observed that straight cracks appear from a strain below 5% and that they multiply during the deformation. Finally, after 8% of strain, blisters are observed which are initiated at the edge of previously created cracks with a stop of the multiplication of the latter, which will be quantified thereafter. Kapton® material is known to deform plastically from 4–5% of macoscopic strain. Actually, the plastic deformation of the substrate should be localized between the film fragments induced by cracks^34^ that may enhance the buckling phenomenon.

**Figure 2:**
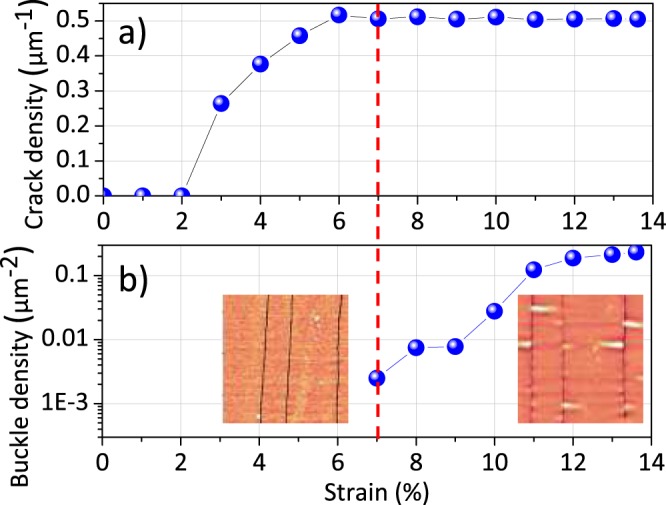
Variations of the crack density (**a**) and of the buckle density (**b**) as a function of the macroscopic deformation applied to the substrate for a 20 nm CoFeB film; 6 × 6 m^2^ AFM images have been inserted for illustration.

Figure 2a shows the evolution of the linear density of cracks as a function of the macroscopic strain applied to the substrate for the 20 nm CoFeB films. It increases from a strain of 2% until saturation after 6%. In addition, it can be seen in 2-b that the surface density of blisters becomes non-zero when the crack density begins to saturate, then increases strongly up to 14%. Thus, these *in situ* experiments show that we are in the presence of two damage regimes: (i) multiplication of cracks and (ii) multiplication of blisters. In order to highlight these two regimes on the same graph, we plotted the surface density of blister *ρ* multiplied by the square of the thickness of the film ${t}_{f}^{2}$ as a function of the linear density of cracks. The choice to multiply by ${t}_{f}^{2}$ is related to the strong effect of thickness on *ρ* and thus makes it possible to compare several thicknesses on the same graph.

**Figure 3:**
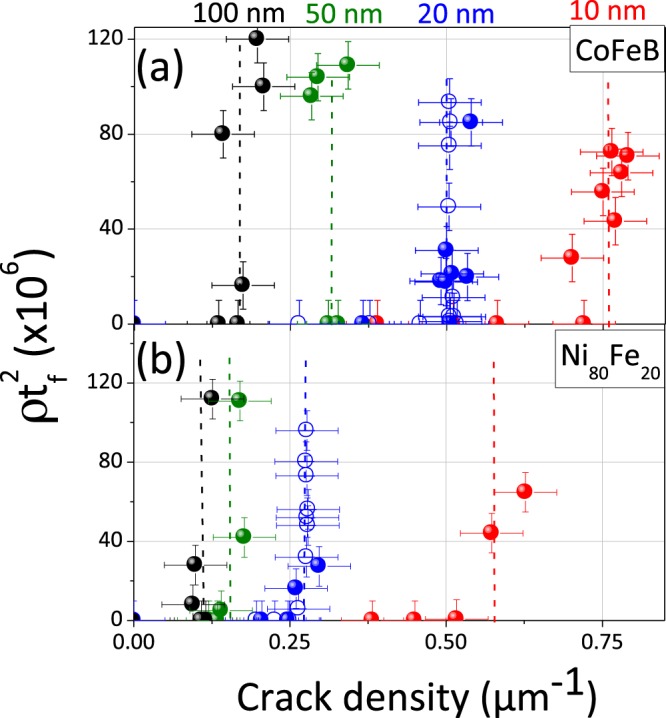
Variations of the surface density of blister *ρ* multiplied by squared thickness of the film as a function of the linear density of cracks highlighting the two different regimes (multi-cracking and buckles). Graph a) corresponds to data for CoFeB films and graph b) to date for Ni_80_Fe_20_ films.

The data corresponding to the previous sample loaded *in situ* is in Fig. 3a) in open blue symbol. It is clear that the parameter $\rho {t}_{f}^{2}$ only increases when the density of cracks reaches saturation of 0.5 m^−1^. For reasons of space, the magnetic measurements whose results are presented in a next paragraph can only be *ex situ* performed. We have therefore undertaken *ex situ* AFM measurements for different maximum strains achieved which we have compared with previously realized *in situ* measurements, for the thickness of 20 nm. Very consistent results are observed (comparison between full and open blue symbols) since the phenomena of cracking and delamination are irreversible.

Further *ex situ* AFM experiments after tensile tests were performed for different thicknesses (10 nm, 50 nm and 100 nm) shown respectively in red, green and black symbols in Fig. 3a). It is observed that the maximum density of cracks decreases with the thickness (from 0.75 m^−1^ for 10 nm to 0.20 m^−1^ for 100 nm), as already described by shear lag models^35^. In addition, the surface density of blisters also decreases with thickness, which results in a slight variation of the term $\rho {t}_{f}^{2}$. In contrast, this behavior has been poorly described in the litterature. Being given that buckling intiate at the edge of cracks, it is obvious that a higher saturating crack density (for thinner films) must lead to higher blister density.

This therefore reflects an increase in the sources of strain heterogeneities when the thickness decreases (greater density of damages) which will have to result in an evolution of the magnetic properties if the magnetoelastic coupling is significant, as will be shown later. This study of the effect of thickness was also carried out for Ni_80_Fe_20_ alloy in Fig. 3b). The same bimodal behavior and the same effect of thickness are observed with, however, slightly lower maximum cracks density values for all the thicknesses. This alloy is also subject to severe damage during uniaxial tests with comparables phenomena.

### Magnetic and magnetoelastic characterization

**Figure 4:**
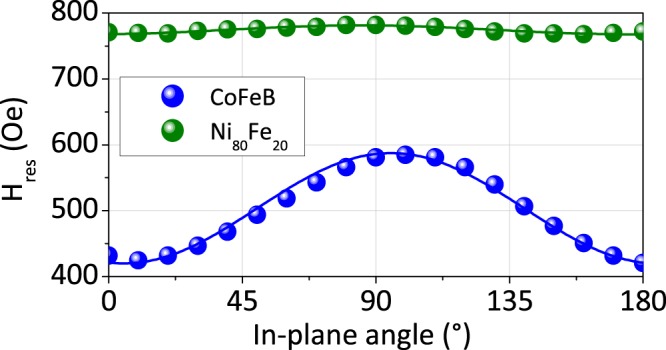
Angular dependence of the resonance field for CoFeB (blue) and Ni_80_Fe_20_ (green) 20 nm-films. Solid lines correspond to fits obtained using Eq. 1 with the parameters indicated in the text.

The “standard” characterization of thin films by magnetic resonance often begins with the measurement of the angular dependence of the resonance field in order to verify the presence of planar anisotropies and their orders^36^. In this regard, Fig. 4 shows the angular dependencies of the resonance field *H*_*res*_ of the 20 nm thick CoFeB and Ni_80_Fe_20_ films measured at 8 GHz. The difference in the average resonance field value for the two materials is simply due to the saturation magnetization difference. In addition, there is the presence of a uniaxial anisotropy (order 2) for the two materials. This residual uniaxial anistotropy *H*_*u*_ can be calculated with $2{H}_{u}={H}_{res}^{max}-{H}_{res}^{min}$. Gueye *et al*.^37^ have shown that in the case of compliant substrates this uniaxial anisotropy is due to the slight curvature of the film/substrate systems subjected to residual stresses. Thus, this anisotropy is weaker as the magnetostriction coefficient is low. Indeed Ni_80_Fe_20_ is known to have a very weak magnetostriction coefficient that is consistent with the very weak measured value of *H*_*u*_ (7 Oe) as compared to one measured for CoFeB (85 Oe). In theses conditions and by asuming that the magnetization is always aligned along the applied magnetic field during the angular rotation, the resonance field angular dependencies can be adjusted using:

1 $${H}_{res}=\sqrt{{(2\pi {M}_{s}+{H}_{u}\sin {\phi }_{H}^{2})}^{2}+{(\frac{2\pi }{\gamma })}^{2}}-2\pi {M}_{s}-{H}_{u}(\frac{1}{2}+\frac{3}{2}\,\cos \,2{\phi }_{H})$$

Where *φ*_*H*_ corresponds to the in-plane angle (referring to the film edges), *M*_*s*_ is the saturation magnetization, *f* is the driving frequency and *γ* is the gryomagnetic ratio. Solid lines in Fig. 4 refer to fits using *M*_*s*_ = 1040 emu cm^−3^ (resp. 755); *γ* = 1.948 × 10^7^ Hz.Oe^−1^ (resp. 1.835 × 10^7^) and *H*_*u*_ = 85 Oe (resp. 7 Oe) for the CoFeB 20 nm-film (resp. Ni_80_Fe_20_). The fitting results are in good agreement with the experimental ones. We have extented this “standard” characterization to all films (all thicknesses). Those measurements show that *M*_*s*_, *H*_*u*_ and *γ* are almost constant as function of the film thickness for both the alloys (see Table 1).

**Table 1:** Saturation magnetization (*M*_*s*_), anisotropy field (*H*_*u*_), gyromagnetic factor (*γ*) and magnetostriction coefficient (*λ*) of CoFeB and Ni_80_Fe_20_ films. nm. means not measured.

Thickness (nm)	*M*_*s*_ (emu cm^−3^)	*H*_*u*_ (Oe)	*γ* (×10^7^) Hz.Oe^−1^	*λ* (×10^−6^)
**Ni**_**80**_ **Fe**_**20**_				
10	750	7	1.835	nm.
20	755	7	1.835	0.72
50	770	6	1.835	nm.
100	790	6	1.835	0.75
**Co**_**40**_ **Fe**_**40**_ **B**_**20**_				
10	1030	74	1.948	22
20	1040	85	1.948	23
50	1080	84	1.948	nm.
100	1095	95	1.948	24

The absolute uncertainty for *λ* is about 0.05 × 10^−6^.

**Figure 5:**
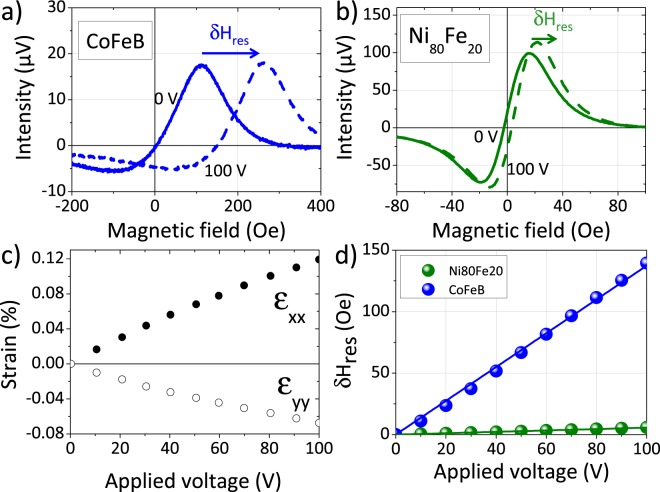
(**a**,**b**) Ni_80_Fe_20_ and CoFeB 20 nm-films resonance spectra for two different voltages applied to the piezoelctronic actuators (0 V and 100 V). (**c**) Variation of the in-plane strains (*ε*_*xx*_ and *ε*_*yy*_) measured at the top surface of the films as a function of the applied voltage d) Resonance field shift (*δH*_*res*_ = *H*_*res*_(100 *V*) − *H*_*res*_(0)) variations as function of the applied voltage. Solid lines refer to the adjusting models for the two samples, using Eq. 2 with the parameters indicated in the text.

In addition, the determination of the magnetostriction coefficient is also an important parameter if one wants to analyze, even qualitatively, the results obtained in the context of large strains (next section). This determination was carried out following the methodology developed in^32^ by making the hypothesis of elastic (Young’s modulus *Y* and Poisson’s ratio *ν*) and magnetoelastic (magnetostriction *λ*) isotropy. For this purpose, we cemented the film/substrate system on a piezoelectric actuator. The principle is to record resonance spectra at different strain states by applying a voltage to the piezoelectric actuator. The transmission of planar strains from the actuator to the thin film is measured by digital image correlation (DIC) and is usually 100% in the case of compliant substrates^31,38^. Figure 5c) gives the correspondance between the applied voltage and the transmitted in-plane strains (*ε*_*xx*_ and *ε*_*yy*_) extracted from DIC measurements. We can see here that *ε*_*xx*_ is positive while *ε*_*yy*_ is negative, the accuracy being less than 10^−5^ in our conditions, as already reported in^38^. Figure 5a,b) show typical resonance spectra of the 20 nm CoFeB and Ni_80_Fe_20_ films at 0 V and 100 V obtained under similar conditions (frequency of 8 GHz and magnetic field applied perpendicular to the actuator, i. e. perpendicular the principal elongation direction). A positive energy shift (*δH*_*res*_) defined as *δH*_*res*_ = *H*_*res*_(100 *V*) − *H*_*res*_(0) of the spectra is clearly observed when a voltage is applied. This shift is less intense in Ni_80_Fe_20_ (around 5 Oe) as compared to CoFeB film (around 140 Oe) and is due to a lower (positive) value of *λ*. The quantitative determination of *λ* is possible by adjusting *δH*_*res*_ evolution as a function of the applied voltage. This evolution is shown in Fig. 5d) for both films. Since the magnetic field is applied parallel to the elongation direction of the actuator, Eq. 1 is only slightly modified^31^:

2 $$\begin{array}{c}{H}_{res}=\sqrt{{(2\pi {M}_{s}+{H}_{u}\sin {\phi }_{H}^{2}+\frac{3\lambda }{2{M}_{s}}{\sigma }_{xx})}^{2}+{(\frac{2\pi }{\gamma })}^{2}}\\ \,\,\,-2\pi {M}_{s}-{H}_{u}(\frac{1}{2}+\frac{3}{2}\,\cos \,2{\phi }_{H})+\frac{3\lambda }{2{M}_{s}}({\sigma }_{xx}-2{\sigma }_{yy})\end{array}$$

*σ*_*xx*_ and *σ*_*yy*_ are the in-plane principal stress tensor components in the films which are simply given by the Hooke’s law. For that purpose, *Y* and *ν* values used for CoFeB and Ni_80_Fe_20_ films have been extracted from refs^39,40^. By adjusting Eq. 2 with the experimental data and knowing physical parameters (see Table 1), we can estimate *λ* with accuracy. The deduced magnetostriction values are 23 ppm for CoFeB and 0.7 ppm for Ni_80_Fe_20_. Note that these values have not been systematically measured for all the other thicknesses but it is known that the thicknesses involved in this work have a negligible influence on the magnetoelastic properties^41^ (see Table 1).

### Large strain effects on magnetic anisotropy

**Figure 6:**
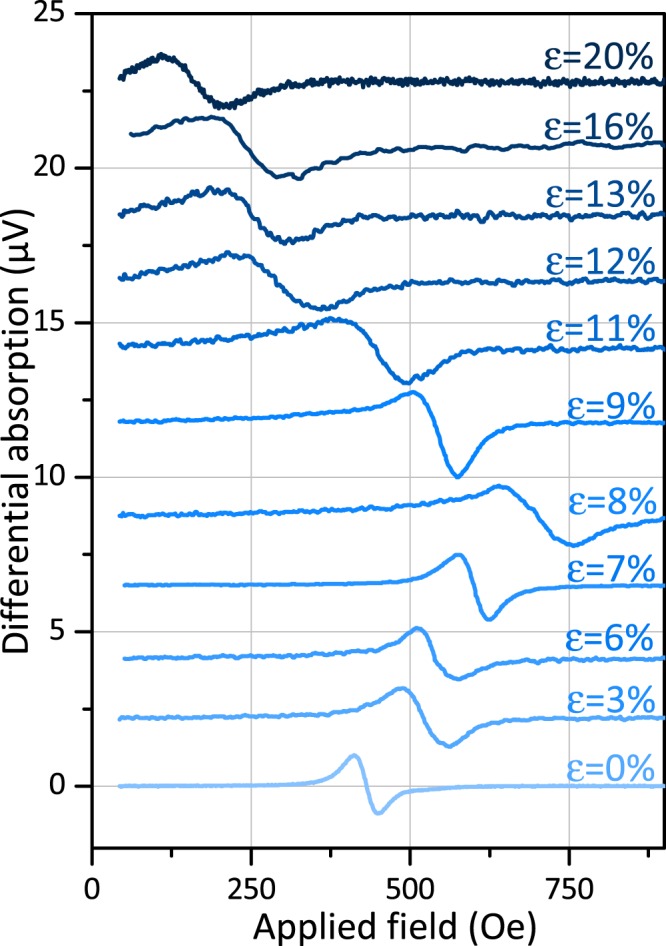
Typical FMR spectra of a 20 nm CoFeB film for different previously applied strains. Spectra have been normalized for clarity. The sketch shows the direction of the applied magnetic field (perpendicular to the pre-applied traction).

**Figure 7:**
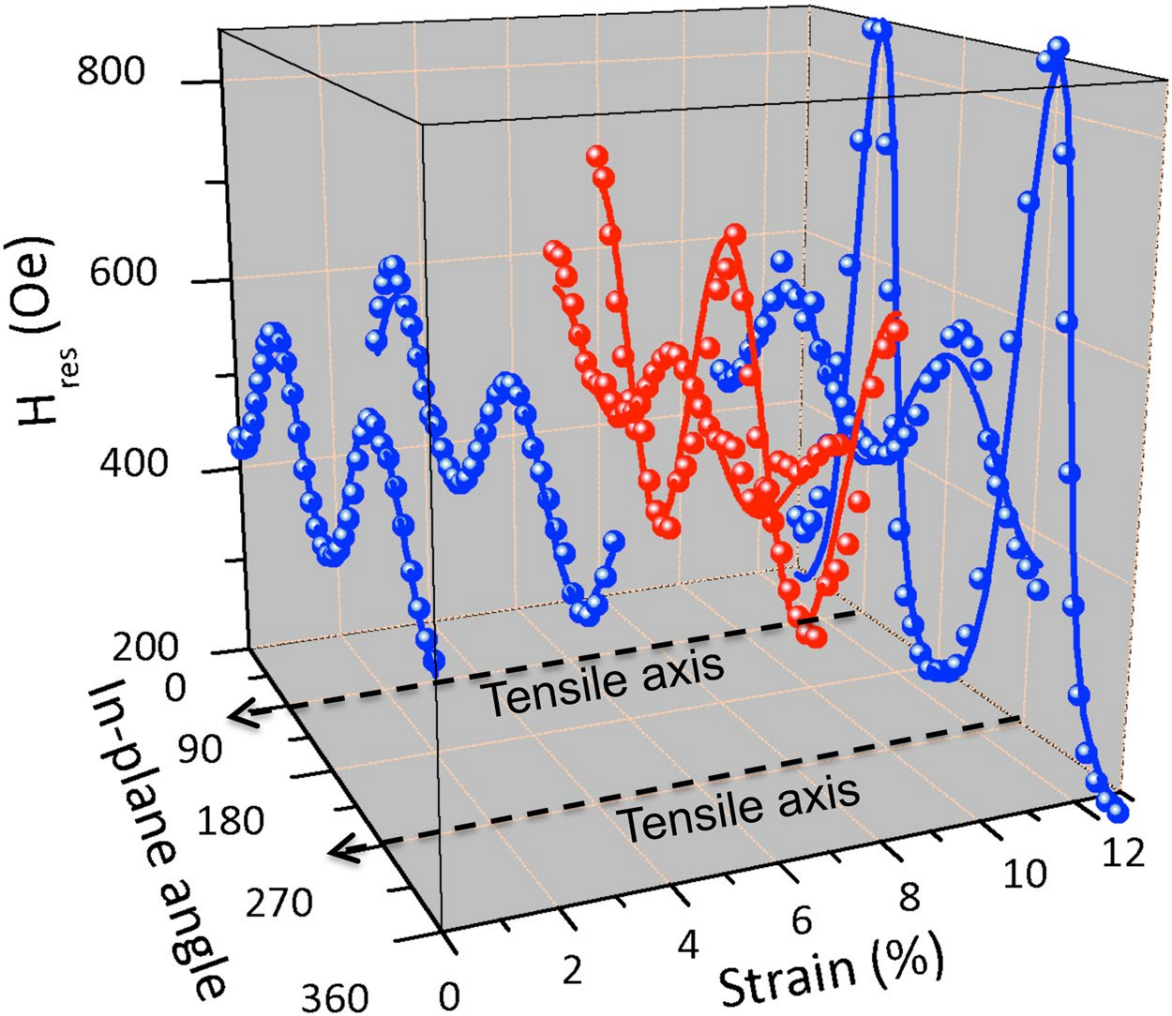
Resonance field (*H*_*res*_) angular dependencies for 20 nm CoFeB films at different previously applied strain. The blue/red color code is used to display the tilting of the direction of the easy axis. Continuous lines refer to the adjusting models for the two samples, using Eq. 1 with *M*_*s*_ and *γ* values previously determined, the only adjustable parameter is *H*_*u*_.

We will now analyze the influence of stretching on the magnetic properties of the studied films. As previously mentioned, it is important to note that FMR measurements have been performed *ex situ*. This is due to geometric constraints, it is indeed not easy to incorporate the tensile machine within the gap of our electromagnet. However, since we are investigating irreversible phenomena, we have analyzed previously deformed *ex situ* films at several levels of deformation. We have thus produced several tensile specimens of the same size and have minimized the time between applied deformation and resonance measurements (a few minutes) to minimize post-test crack closure phenomena. We have performed angular dependence measurements in order to follow the evolution of planar magnetic anisotropies. The driving frequency is fixed at 8 GHz throughout this section. We first present results from CoFeB films. Typical FMR spectra of 20 nm CoFeB films are presented in Fig. 6. These spectra are obtained by applying a magnetic field transversely to the traction previously applied. It is remarkable to note that even after the application of large strains (up to 20%), the signal is preserved. Moreover, we realize that the resonance field evolves strongly from one spectrum to another. This is due to the variation of the planar anisotropy of the films. Thus, Fig. 7 shows the angular dependencies of the resonance field for different strains applied to 20 nm CoFeB films. In these angular dependencies, the tensile stress is applied at 90°. In absence of strain (0%), the easy axis is at 0°. Beyond 6%, the easy axis is found along the applied stress. We used a red/blue color code to illustrate this directional tilting of the easy axis. Beyond 11%, the easy axis returns to 0°. Moreover, in addition to the tilting of this axis of easy magnetization from 0° to 90° and then again to 0°, it is noted that the value of the anisotropy field also varies.

**Figure 8:**
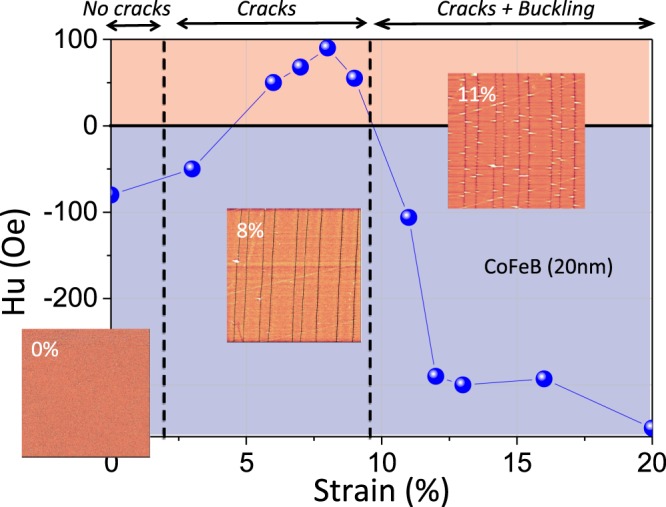
Variation of *H*_*u*_ as a function of the strain for 20 nm Co_40_Fe_40_B_20_ films. *H*_*u*_ is counted positively when it is aligned along the traction and negatively when it is perpendicular to it. The AFM images (20 × 20 mm^2^) presented are characteristic of the three regimes.

Figure 8 represents the variation of the deduced uniaxial anisotropy field *H*_*u*_ as function of the applied strain. We used the following convention for the sign of this field: it is positive when the easy axis is along the traction and negative when this axis is at 90° of the traction. This curve illustrates the results obtained from the angular dependences of the resonance field. It is clear that it is initially negative, then positive between 5% and 10% and then again negative beyond 11%.

In order to explain this complex behavior with strain, a first assumption could be the effect of a shape anisotropy linked to the parallel nature of cracks between them. Indeed, if we see this set of fragments as an array of nanowires, this could explain the shift towards negative values^42^. However, the precedent subsection shows that the appearance of cracks occurs at less than 2% of deformation and the saturation of the width of the fragments is obtained at 6% of strain, values for which *H*_*u*_ is still positive, which tends to invalidate this hypothesis. Moreover, an elementary magnetostatic calculation^43^ shows that such a shape anisotropy field is significant only for lateral dimensions smaller than those of our fragments (rather a hundred nanometers whereas our fragments have a width of about 2 mm).

Furthermore, solid lines in Fig. 7 are fits to the experimental data. These fits have been plotted using Eq. 1 and by using *M*_*s*_ and *γ* values of the unstrained state (see Table 1), the only adjutable parameter is *H*_*u*_. The good agreement between fits and experimental data shows that *M*_*s*_ and *γ* values are not affected by large strains and subsequent fragmentation of the films. In these conditions, we have no reason to think either that the magnetostriction coefficient *λ* changes strongly or that it changes sign.

We have to find a better correlation with the results obtained in the precedent subsection. We have highlighted the presence of 3 distinct regimes for these CoFeB films: (i) elastic domain, (ii) crack multiplication, (iii) decohesion with fixed crack density. Figure 8 shows which ranges of strains correspond to these regimes. We also show a typical AFM image (20 × 20 mm^2^) of each of these regions. We will discuss the *H*_*u*_ variations, based on the planar stress state induced by each tensile test. Indeed, we know that the magnetoelastic contribution to the anisotropy field can be simply written as a function of planar stresses^32^:

3 $${H}_{u}=\frac{3\lambda }{{M}_{s}}({\sigma }_{xx}-{\sigma }_{yy})$$

We are in presence of *ex situ* measurements. Thus, any magnetoelastic contribution to the anisotropy field can only be due to residual stresses generated during the tensile test and during the elastic discharge of the substrate. First, it is clear that the multiplication of cracks is correlated with the first *H*_*u*_ change of sign. Indeed, for fragile films, it is known in this regime that a longitudinal stress plateau is observed while a transverse compressive stress develops and increases as long as no decohesion of the film occurs. This phenomenon is illustrated in Fig. 7 of ref.^34^ for Ta-*α* films for which the stresses are *in situ* measured by x-ray diffraction. We believe that this difference between *σ*_*xx*_ and *σ*_*yy*_ is sufficiently important for reminiscence in the residual state after discharge. This explains the positive sign of *H*_*u*_ before onset of decohesion. After appearance of first decohesions, the transverse stress of compression decreases strongly in magnitude while the longitudinal stress of tension decreases and can tend towards zero for strains of 20% as also shows in Fig. 7 of ref.^34^. Then, a discharge of the substrate from such a state of stress in the film can quite reverse the sign of transverse and longitudinal stresses. Since we have not identified any other source of modification for a magnetoelastic contribution, we believe that such a reversal of the stresses has occurred.

**Figure 9:**
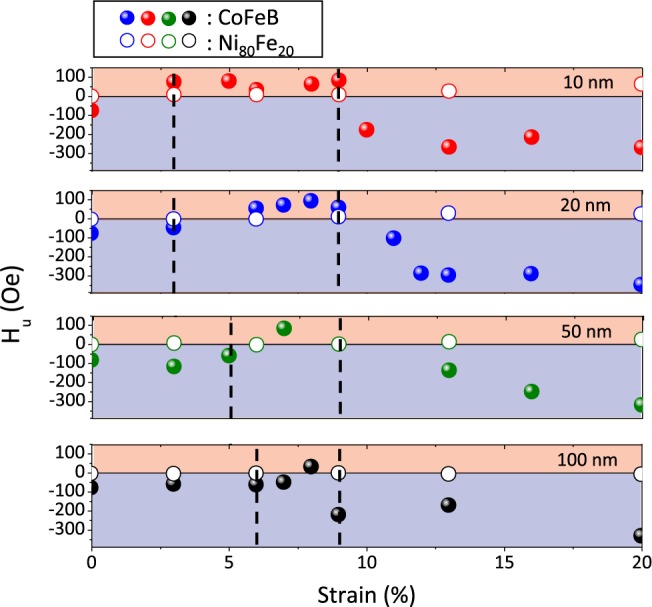
*H*_*u*_ variations as a function of the applied strains for CoFeB (filled symbols) and Ni_80_Fe_20_ (open symbols) films of different thicknesses: 10, 20, 50 and 100 nm. This field is counted positively when it is aligned along the traction and negatively when it is perpendicular to it. The Co_40_Fe_40_B_20_ films regimes (“absence of cracks”, “cracks” and “cracks + buckling”) are delimited by vertical black dotted lines.

In order to confirm our observations on these 20 nm CoFeB films, we performed tensile tests followed by magnetic resonance measurements on the other CoFeB films (10, 50 and 100 nm). The results from angular dependence measurements are presented in Fig. 9. In this figure, the dashed black vertical lines delimit the three regimes that we have identified through *in situ* AFM observations. It is immediately interesting to note that the regime “cracks + buckling” starts approximately for the same strain (~9%) while “crack initiation” regime is dependent on the thickness of the film. This initiation starts at ~2% for films of 10 and 20 nm, at ~4% for 50 nm films and at ~6% for 100 nm films. We note that the first sign inversion of *H*_*u*_ is dependent on the films thickness. The strain for which this inversion appear increase with the films thickness: ~3–4% for 10 nm; at ~5–6% for 20 nm; at ~6–7% for 50 nm and at ~7–8% for 100 nm. This inversion is strongly correlated with the appearance of the first cracks which is also dependent on the thickness. There is an inertia between the appearance of the cracks and the first sign inversion for each thickness This is not surprising since it is probably necessary to achieve a sufficient density of cracks to generate the state of residual stresses necessary to change the *H*_*u*_ sign.

On the other hand, it is interesting to note that the second sign inversion takes place roughly for the same strains whatever the thickness, which again is well correlated with the delimitation between the “crack” and “cracks + buckling” regimes. Finally, concerning the *H*_*u*_ value, we note that the minimum and maximum values of *H*_*u*_ are approximately same (regardless of the strains for which sign inversions occur) whatever the thickness and therefore the width of the fragments. Moreover these values of the order of 300–400 Oe are much higher than those expected for the possible shape anisotropy which definitively exclude this hypothesis. We conducted similar experiments with Ni_80_Fe_20_ films. Their *H*_*u*_ variations are presented in Fig. 9 (open symbols). One can note here that these *H*_*u*_ values come from adjustments of the angular dependencies of *H*_*res*_. As for CoFeB, *M*_*s*_ and *γ* values do not seem to be affected by these large deformations. We observe that *H*_*u*_ is almost constant for Ni_80_Fe_20_ films even if similar damages (multicracking and buckling) are also observed. Thus, we attribute this monotony of *H*_*u*_ as a function of strains to the almost zero magnetostriction coefficient for this alloy, thus confirming that these results must be analyzed in the context of residual stresses and that contributions other than magnetoelastic could be neglected (such as magnetostatic contributions due to breaking translation invariance).

### Large strain effects on magnetic damping

**Figure 10:**
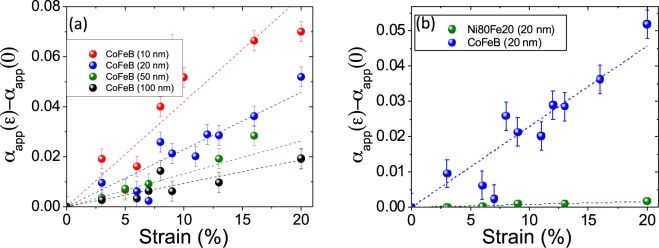
(**a**) Evolution of the apparent damping *α*_*app*_ as a function of the previously applied strains for CoFeB films. (**b**) Variation of *α*_*app*_ as a function of the previously applied strains for 20 nm films of both materials (CoFeB and Ni_80_Fe_20_).

Finally, we have used the spectra recorded at 8 GHz to analyse the evolution of the magnetic damping parameter, which is of strong importance in spintronic applications since it governs the speed at which the magnetization can be reversed or reoriented. In a fixed frequency field-swept FMR experiment, the peak to peak linewidth Δ*H*_*pp*_ is proportional to the frequency with a slope determined by^44,45^:

4 $${\rm{\Delta }}{H}_{pp}={\rm{\Delta }}{H}_{0}+\frac{2\alpha }{\sqrt{3}\gamma }2\pi f$$

where Δ*H*_0_ is referred to inhomogeneous contributions to the linewidth (that can be also frequency dependent^46^) and *α* is the magnetic damping. This equation is only valid when the magnetization is aligned along the applied magnetic field, which is almost the case for our analyzed spectra. In the following; for the sake of brevity, we define an “apparent” damping parameter *α*_*app*_ deduced from the mean values of Δ*H*_*pp*_ as function of the in-plane angle (at 8 GHz). As we are interested in the evolution of the apparent damping as function of the strain, we have plotted *α*_*app*_(*ε*) − *α*_*app*_(0) (where *α*_*app*_(0) is the value of the unstrained films) as function of the pre-applied strain in Fig. 10a) for CoFeB films. Figure 10b compares the *α*_*app*_ evolution for the two studied alloys (20 nm films). The *α*_*app*_ parameter is the signature of the heterogeneities present within the films. Thus it will depend on the strains heterogeneities and will be exacerbated by the average strain. In term of magnetism, it corresponds to the heterogeneity of *H*_*u*_ exacerbated by its average value. Note that this coefficient has a globally increasing dependence for both materials as function of the strain. The dotted lines are there to mark this trend. For CoFeB films, we note that the greater the thickness, the less the slope of these lines is important (see Fig. 10a)). This is consistent with the previous results of this work. Indeed, the thinnest films have cracks densities and decohesions the most important. They are sources of strong heterogeneities. We remember that the evolution of the average value of *H*_*u*_ was rather weakly dependent on the thickness, it is therefore a pure effect of heterogeneity. In fact, if these increases were due only to an increase (in absolute value) of the average anisotropy field, the final values of *α*_*app*_ would all be the same at 20% of deformation, which is not the case. In addition, in these evolutions, we note the influence of the contribution of the average value of *H*_*u*_. Indeed, if we look closely at the CoFeB curve, we can see the signature of the “cracks” region that we observed for *H*_*u*_ (softening of *α*_*app*_ between 2% and 7% that we see particularly well for 10 nm and 20 nm films).

For the Ni_80_Fe_20_ films, we notice that high strains have only small relative influence on *α*_*app*_ values. This is quite remarkable when one puts these results in relation to the multiple fissures and decohesions present within these films. Indeed, the low magnetostriction makes this material insensitive from a magnetic point of view to the strains heterogeneities. This conclusion is highlighted by Fig. 10b) where we clearly see the small effect of these large strains on *α*_*app*_ compared to CoFeB films.

## Conclusion

Two materials commonly used in the field of spintronics (Co_40_Fe_40_B_20_, Ni_80_Fe_20_) have been deposited on a Kapton® substrate, with variable thicknesses (from 10 nm to 100 nm). We have shown that these films have two distinct irreversible deformation regimes, the first being related to the multiplication of cracks and the other to the multiplication of blisters. Overall, the density of these damages is increasing when the thickness decreases. Subsequently, we identified the *ex situ* effects of large strains and particularly of the “cracks” and “cracks + buckling” regimes. In the case of Co_40_Fe_40_B_20_ films, the magnetic anisotropy field undergoes two sign changes, the first being attributed to the residual stress field related to the crack distribution and the second to the strong transverse stress relaxation when buckling occurs. In contrast, the anisotropy remains stable for Ni_80_Fe_20_, which is attributed to its low magnetostriction coefficient. Moreover, one notes a strong increase of the damping as a function of the macroscopic strain applied to Co_40_Fe_40_B_20_/Kapton® systems. This was very little observed for the Ni_80_Fe_20_. Indeed, magnetic damping is directly related to the magnetic field heterogeneities in the material. In this case, this is the magnetoelastic field heterogenity, related to strain heterogenities due to damages, which induces the increase of damping. Thus, a material which magnetic properties are insensitive to strains by nature has the remarkable property of behaving in a substantially similar manner whether it is “intact” or exhibiting high densities of cracks and blisters. This is undoubtedly due to the fact that the inter-cracks distance and the buckles size are a little too high to induce significant anisotropies linked to geometrical features.
